# Optical Imaging Visualizes a Homogeneous and Horizontal Band-Like Biodistribution of Large- and Small-Size Hydrophilic Compounds Delivered by Ablative Fractional Laser

**DOI:** 10.3390/pharmaceutics14081537

**Published:** 2022-07-23

**Authors:** Rikke Louise Christensen, Vinzent Kevin Ortner, Merete Haedersdal, Uffe Høgh Olesen

**Affiliations:** Department of Dermatology, Copenhagen University Hospital, Bispebjerg and Frederiksberg, 2400 Copenhagen, Denmark; vinzent.kevin.ortner@regionh.dk (V.K.O.); mhaedersdal@dadlnet.dk (M.H.); olesen.uh@gmail.com (U.H.O.)

**Keywords:** laser-assisted drug delivery, ablative fractional CO_2_ laser, low pulse energy, dermatology, skin, antibody, PD-1 inhibitor, nivolumab, ex vivo confocal microscopy, biodistribution

## Abstract

The skin barrier generally limits the topical delivery of hydrophilic molecules. Ablative fractional laser (AFL) facilitates cutaneous drug uptake of smaller hydrophilic compounds in several studies. In this imaging-based study, we aim to investigate the cutaneous biodistribution of two different-sized hydrophilic compounds delivered by an ablative fractional CO_2_ laser at minimally invasive settings. Intact or CO_2_ AFL-pretreated (2.5 mJ/mb and 5% density) ex vivo porcine skin was topically applied with a large or small hydrophilic compound (fluorescence labeled antibody nivolumab (150,000 g/mol, *n* = 4) or ATTO 647N (746 g/mol, *n* = 3)). Samples were incubated for 20 h in a Franz cell setup, whereafter optical coherence tomography (OCT) was used to assess laser channel depth, and ex vivo confocal microscopy (EVCM) was used to assess epidermal thickness and cutaneous biodistribution of nivolumab and ATTO 647N. With an EVCM-assessed median epidermal thickness of 70.3 µm and OCT-assessed ablation depth of 31.9 µm, minimally invasive settings enabled shallow penetration into the mid-epidermis. The AFL-assisted uptake of the antibody nivolumab and the smaller compound ATTO 647N showed a similar homogenous and horizontal band-like biodistribution pattern that reached mid-dermis. No uptake of nivolumab or ATTO 647N was observed in intact skin. In conclusion, AFL-induced mid-epidermal laser channels facilitates the cutaneous delivery of two hydrophilic compounds that are distributed in a similar homogeneous and horizontal band-like pattern, irrespective of their molecular size.

## 1. Introduction

A main function of the skin is to provide a protective barrier against environmental insults. The barrier is composed of the outermost stratum corneum. consisting of densely packed corneocytes embedded in a matrix of hydrophobic non-polar lipids. The ability of a drug to permeate the skin is influenced by its hydrophilicity and molecular weight. While hydrophilic drugs have poor penetration properties and need assistance to cross the skin barrier, lipophilic agents are more likely to pass through the skin’s hydrophobic upper layers. Similarly, molecules over 500 Da penetrate intact skin sparingly [1,2,3,4].

Ablative fractional lasers (AFL) have been shown to significantly enhance cutaneous uptake of various topically applied drugs [5,6,7,8]. Via microscopic channels created by the laser, the skin barrier is disrupted and a route for cutaneous drug uptake is achieved [1]. Several parameters can influence laser-assisted drug delivery (LADD), including physicochemical drug properties, incubation time, and laser settings. Laser pulse energy (mJ/mb) controls the depth of the laser channels—the higher the pulse energy, the deeper the channels. Deeper channels may be an advantage for the delivery of hydrophilic molecules as the hydrophilic molecules have a greater solubility in the aqueous environment in the laser channels [1]. In some studies which investigate the delivery of hydrophilic molecules, deeper channels have resulted in higher drug concentrations throughout the skin [5,7,9]. In addition, smaller molecules (400–1000 Da) have a tendency to result in greater dermal uptake compared to larger molecules (2000–3350 Da) delivered using identical laser settings [3].

The interest in laser-assisted delivery of very large molecules such as antibodies (150 kDa) has increased in the past 5 years [6,10,11,12]. Topical delivery of therapeutic antibodies could be an advantage for the treatment of various skin diseases, providing a topical, targeted treatment compared to systemic administration, whereby possible side effects might be avoided. Three LADD studies successfully demonstrated the delivery of antibodies using an Er:YAG laser [6,11,12] and two studies from our lab showed delivery of the anti-PD-1 antibody nivolumab with an ablative fractional CO_2_ laser [13,14]. Further, a recent study showed reduced tumor growth in mice after treatment with an ablative fractional CO_2_ laser, followed by topical application of anti-PD-1 antibody coated patches, indirectly indicating a successful delivery of the anti-PD-1 antibody into mouse tumors [10]. Although there are clear advantages of using low pulse energy and density, including reduced bleeding, oozing, and pain, as well as faster healing [1], assessments of AFL using low settings in the context of antibody delivery is sparse.

The aim of this imaging-based study is to visualize the cutaneous biodistribution of two hydrophilic compounds, the antibody nivolumab (150 kDa), and the fluorescent molecule ATTO 647N (746 Da), delivered by an ablative fractional CO_2_ laser at low pulse energy and low density.

## 2. Materials and Methods

### 2.1. Study Setup

Cutaneous ex vivo delivery of two differently sized molecules was tested with an ablative fractional CO_2_ laser at low pulse energy and low density. The study included four interventions (*n* = 3–4 per intervention): intact- or AFL-treated skin with topical application of either fluorescently labelled nivolumab (Bristol Myers Squibb, New York City, NY, USA) or ATTO 647N (ATTO-TEC GmbH, Siegen, Germany). Following 20 h of topical drug exposure, the cutaneous uptake was investigated by EVCM. OCT was incorporated for a non-invasive measurement of laser channel depth.

### 2.2. Skin Source

In the study, we used porcine skin as it is the most common substitute for human skin. Discarded porcine skin from the flank of two female pigs (Landrace, Yorkshire, and Duroc mixed race, at 34 kg and 57 kg) was obtained from University of Copenhagen, Department of Experimental Medicine. The skin was trimmed for hair and subcutaneous fat. Squares of 3 × 3 cm skin were excised and stored at −80 °C until study initiation (up to three months maximum). Skin was thawed at room temperature before treatment.

### 2.3. Molecule Preparation

Nivolumab (Bristol Myers Squibb, New York City, NY, USA), an anti-PD-1 antibody, was tagged with HiLyte Fluor^TM^ 488 SE using the protocol of the manufacturer (λ_Abs_ = 499 nm, λ_em_ = 523 nm) (AnaTag^TM^ HiLyte Fluor^TM^ 488 Protein labeling Kit, AnaSpec, Inc, Fremont, CA, USA) and diluted in distilled water to a concentration of 1 mg/mL.

The fluorescence molecule ATTO 647N (free acid modification, λ_Abs_ = 646 nm, λ_em_ = 664 nm) (ATTO-TEC, Siegen, Germany) was dissolved in distilled water 0.05% DMSO to a concentration of 10 µM.

### 2.4. Laser-Assisted Drug Delivery

For this study, we used a commercially available ablative fractional 10,600 nm CO_2_ laser (Ultra-pulse, DeepFx handpiece, Lumenis Inc., Yokneam, Israel), commonly used in dermatology, with which we have previously shown LADD of antibodies using higher energies [13,14]. Areas of 10 × 10 mm skin were exposed to the lowest device-specific pulse energy of 2.5 mJ/mb at 150 Hz and 5% density, which has been reported as most relevant in LADD of hydrophilic molecules [3].

Immediately after AFL treatment, the skin was coupled to a Franz cell setup, as previously described (PermeGear Inc., Hellertown, PA, USA) [15]. Receiver chambers were loaded with approximately 5.5 mL 1xPBS (pH 7.4, with no further addition of any solvent) and donor chambers with exactly 0.5 mL nivolumab or ATTO 647N solution. The skin was detached from the Franz cell after 20 h, and 8 mm punch biopsies were collected and stored at −80 °C.

### 2.5. Ex Vivo Confocal Microscopy

Vertical skin sections of 100 µm were cut from the biopsies and mounted on a bimodal EVCM (Vivascope^®^ 2500, MAVIG GmbH, Munich, Germany) for combined reflectance (RCM, λ_em_ 638 nm) and fluorescence microscopy (FCM, λ_em_ 488 nm for nivolumab and 638 nm for ATTO 647N) [16]. The EVCM uses a 38X water–gel immersion objective that, together with other optics, will give a final microscope magnification of 550×. RCM images were captured to visualize cutaneous micromorphology and measure epidermal thickness (*n* = 42 measurements in 14 biopsies). For FCM imaging, the lasers were adapted to the fluorophore’s quantum yield, using 100% fluence for nivolumab and 80% fluence for ATTO 647N. FCM images were assessed for biodistribution and signal intensity.

### 2.6. Optical Coherence Tomography

Channel depth (*n* = 18 channels in 6 biopsies) was measured using a commercially available swept-source optical coherence tomography (OCT) (VivoSight^®^ Dx, Michelson Diagnostics Ltd., Kent, UK) [17]. The OCT is equipped with an infrared laser (1305 nm), which provides a penetration depth of up to 1500 µm and an optical resolution of <7.5 µm laterally and <5 µm axially. For each biopsy, we captured a 250-frame scan.

### 2.7. Image Analysis and Statistics

In EVCM images, we quantitated the mean fluorescence intensity (MFI), defined as the average fluorescence signal intensity (arbitrary unit, AU) of FCM images and measured the depth (µm) of the fluorescence signal as the distance between the skin surface and loss of fluorescence signal. For OCT-assessed channel morphometry, we defined channel depth (µm) as the perpendicular distance between skin surface and the deepest point of the ablation crater. EVCM images and OCT scans were analyzed using Fiji ImageJ 1.49 (National Institute of Health, Bethesda, MD, USA) [18]. Descriptive statistics, including medians with interquartile ranges (IQR), were performed in SPSS Statistics version 25 (IBM Corp, Armonk, NY, USA) to describe epidermal thickness, channel depth, fluorescence intensity, and depth of the uptake. In addition, a Mann–Whitney test was performed on fluorescence signal intensity measurements, comparing intact skin with AFL-treated skin with significance level at *p* < 0.05.

## 3. Results

### 3.1. Laser-Tissue Interaction

**Figure 1:**
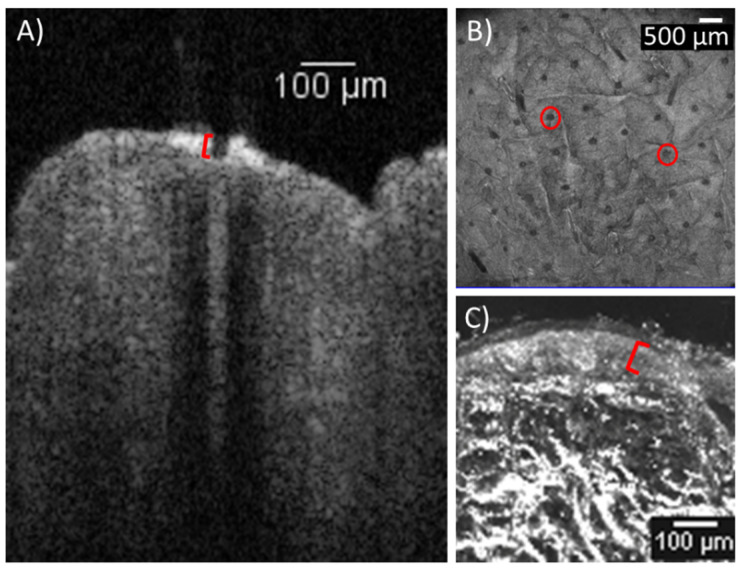
Laser channels and epidermis. Porcine skin treated with ablative fractional CO_2_ laser at 2.5 mJ/mb, 5% density and 250 Hz at an area of 10 × 10mm. (**A**) Vertical OCT image showing an example of a laser channel (marked in red). The median depth of the channels reached 31.9 µm into the epidermis (*n* = 18). (**B**) Horizontal OCT image of the skin surface showing the grid of laser channels penetrating the skin (examples marked in red). (**C**) Ex vivo confocal microscopy image displaying close-up of the epidermis (marked in red). Epidermal thickness was measured to a median of 70.3 µm (*n* = 42). OCT: optical coherence tomography.

Morphometric assessment of the AFL-treated skin using OCT showed laser channels penetrating into the mid-epidermis. The laser channels measured a median depth of 31.9 µm (IQR: 19.9–37.8 µm, Figure 1), while the epidermis measured in the EVCM images showed a median thickness of 70.3 µm (IQR: 64.2–79.0 µm).

### 3.2. Cutaneous Biodistribution

**Figure 2:**
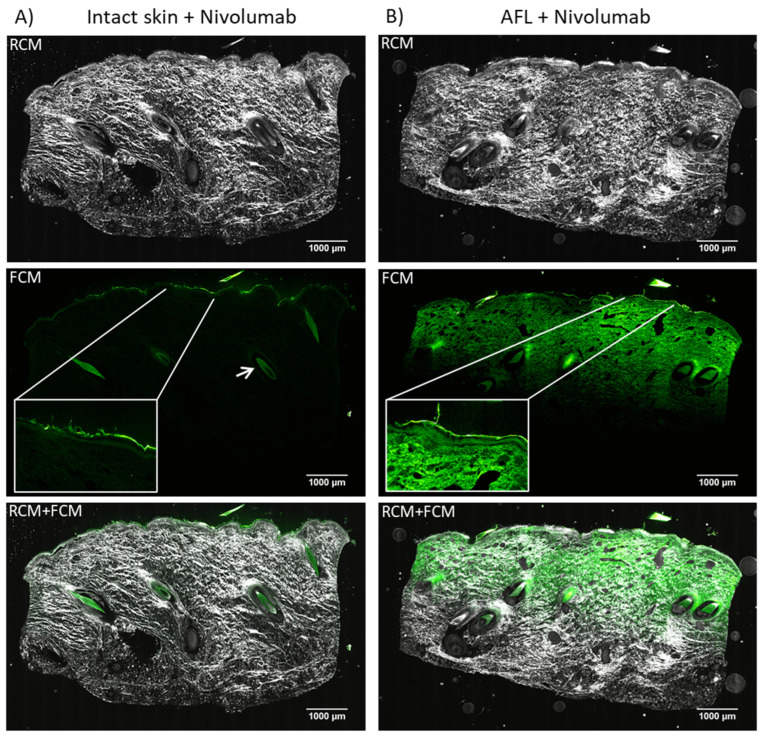
Cutaneous biodistribution of nivolumab (150 kDa). Imaging using ex vivo confocal microscopy of ex vivo porcine skin exposed to 20 h passive diffusion in a Franz cell setup. (**A**) Vertical tissue sections of intact skin exposed to fluorescently (HiLyte Fluor 488 SE) labeled nivolumab (*n* = 3). Remnant was observed on the skin surface (box with close-up) as well as autofluorescence throughout dermis and from the hair follicles in particular (arrow). (**B**) Vertical tissue sections exposed to AFL followed by topically applied fluorescently labeled nivolumab (*n* = 3). Imaging of nivolumab biodistribution showed a homogenous, horizontal band-like uptake that faded with depth. The uptake reached mid-dermis measured to a median tissue depth of 1967 µm. No accumulation of nivolumab was seen in any specific tissue compartment. LUT in ImageJ: Green Hot. AFL: ablative fractional laser. RCM: Reflectance mode. FCM: Fluorescent mode.

Visualization of AFL-assisted delivery of nivolumab revealed a homogenous, horizontal band-like cutaneous uptake in all samples. The fluorescence intensity faded with increasing depth into the skin, and reached mid-dermis measured to a median depth of 1967 µm (IQR: 1939–1980 µm, Figure 2B). No accumulation of nivolumab was seen in neither epidermis nor dermis. Images of intact skin showed no uptake (Figure 2A), and only a dim fluorescent signal from remnant nivolumab was visible on the skin surface of the intact skin samples. Furthermore, autofluorescence was visible throughout dermis of the intact skin with peak signal intensity in the hair follicles. MFI of the entire EVCM image showed a median of 2783 AU (IQR: 2544–2838 AU, Table 1) for AFL-treated skin images, while intact skin samples exposed to nivolumab revealed a lower median MFI of 1731 AU (IQR: 1715–1765 AU); however, this is not statistically significantly different (*p* = 0.100).

**Figure 3:**
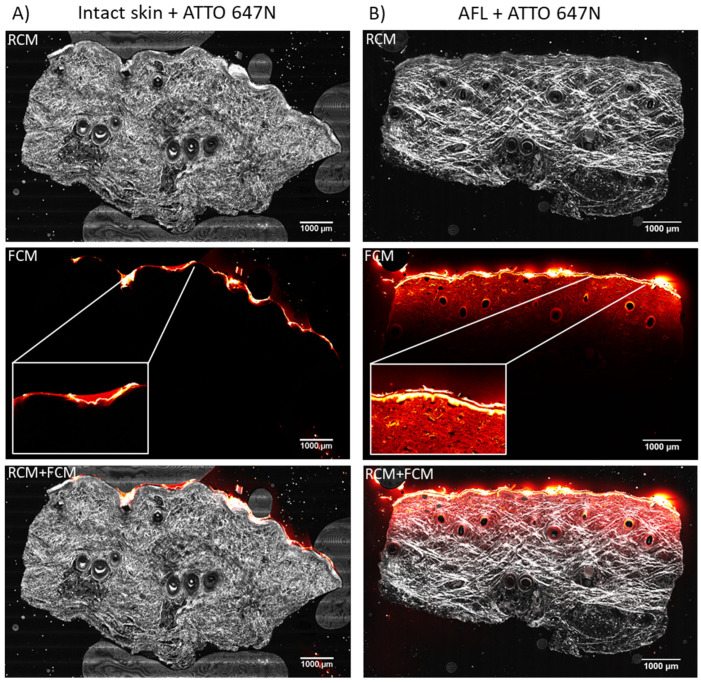
Cutaneous biodistribution of ATTO 647N (746 Da). Ex vivo confocal microcopy imaging of ex vivo porcine skin after 20 h passive diffusion in a Franz cell setup. (**A**) Vertical tissue sections of intact skin exposed to ATTO 647N (*n* = 4). Images showed no uptake into the intact skin, but remnants of ATTO 647N on the skin surface (box with close-up) was clearly visible. (**B**) Tissue sections of AFL-treated skin with topically applied ATTO 647N (*n* = 4). A homogenous, horizontal band-like dermal uptake that decreased with depth and reached a median tissue depth of 1317 µm was seen. In addition, an increased ATTO 647N concentration in epidermis was observed in the AFL-treated skin (box with close-up). LUT in ImageJ: Red Hot. AFL: ablative fractional laser. RCM: Reflectance mode. FCM: Fluorescent mode.

The biodistribution of ATTO 647N in AFL-exposed skin was similar to nivolumab, distributing in a horizontal band across the entire sample. (Figure 3B). A decrease in fluorescence intensity with depth and an increased deposition of ATTO 647N in the epidermis compared to the dermis were visible. The laser-assisted cutaneous uptake was detectable to a median depth of 1317 µm (IQR: 1269–1353 µm). Aside from remnants of ATTO 647N on the skin surface, no uptake of ATTO 647N in intact skin was observed. The AFL-treated skin had a median MFI measured in the entire image of 2727 AU (IQR: 2436–3076 AU, Table 1). Imaging of intact skin exposed to topical applied ATTO 647N revealed a median MFI of 1911 AU (IQR: 1811–2048 AU), which was significantly lower compared with AFL-treated skin (*p* = 0.029, Table 1).

## 4. Discussion

In this study, we found that the two hydrophilic molecules, nivolumab and ATTO 647N, were successfully delivered by LADD using minimally invasive settings. Despite a 200-fold size difference, the two molecules diffused into the skin in a similar biodistribution pattern, shown by EVCM. Nivolumab and ATTO 647N both accumulated in a homogenous, horizontal band-like pattern reaching mid-dermis, which suggests that biodistribution of hydrophilic molecules may be independent of molecular size. Although the results of this study proved the concept of low-energy LADD, more research is needed to determine if laser-assisted delivery of therapeutic antibodies using low settings can exert a sufficient treatment effect. In a preclinical study by Cao et al., anti-PD-1 antibodies were successfully delivered into mouse tumors using a CO_2_ laser. The study resulted in a tumor size reduction that indirectly showed successful delivery of the antibodies [10]. This is a promising result for further investigation in LADD of therapeutic antibodies.

An ablative fractional CO_2_ laser at 2.5 mJ/mb and 5% density was used in our study. Our group has previously shown successful topical delivery of nivolumab into porcine skin using an ablative fractional CO_2_ laser, though at much higher pulse energies than the 2.5 mJ/mb in the present study (20 mJ/mb and 80 mJ/mb) [13,14]. However, both the current and previous works report a similar homogenous diffusion pattern in epidermis to mid-dermis. In one of the studies, a slight increase was seen in the coagulation zones [14]. In the images in the present study, it is not possible to detect the coagulation zones, presumably due to the low pulse energy causing comparatively smaller MTZ, as well as the molecular size. Previous studies on smaller molecules suggest the molecules to accumulate in the coagulation zones, from where they then diffuse into the surrounding tissue [19,20]. Other studies have succeeded in topical delivery of antibodies using Er:YAG lasers [6,11,12]. Here, the antibodies diffused from the channels into the dermis after 24 h [11,12], comparable to our 20 h application time. Measurements at earlier time points of 0–12 h showed localization of the antibodies in the rim or coagulation zone of the channels [6,12]. Thus, even though Er:YAG lasers create thinner coagulation zones compared with ablative fractional CO_2_ lasers, large antibodies may diffuse into the coagulation zones from where they later distribute throughout the skin [21,22].

We used a low pulse energy of 2.5 mJ/mb to investigate the delivery of large hydrophilic molecules. Low pulse energy, compared to high pulse energy, creates superficial channels which could reduce the healing time of the skin as well as the bleeding and oozing from the channels, which may increase the accessibility for the drug and decrease its washout [1,23]. The measured laser channel depth of 31.9 µm did not penetrate through the epidermis, which indicates that the epidermis is not a barrier for the uptake of hydrophilic molecules. Studies using a fractional CO_2_ laser at a pulse energy of 2.5 mJ/mb for drug delivery are scarce. One recent study investigated the delivery of the fluorescent molecule indocyanine green (ICG) with a fractional CO_2_ laser also using 2.5 mJ/mb and 5% density. Even though they used ex vivo human skin [24], it was shown that drug biodistribution among human and pig skin is similar [25]. In their study, they observed a deeper laser channel depth of 70 µm using OCT. The epidermal thickness was not measured in the ICG-delivery study, but a recent study found a median depth of 76 µm in abdominal human skin [25]; thus, the channels might not penetrate through the epidermis similar to our study on pig skin. The different channel depths could be due to differences in the hydration of the skin, the resolution of the OCT or deviations in the laser beam angle to the focal plane of the skin, which has been shown to have a great impact on the laser channel morphometrics [26].

ATTO 647N was detected to a depth of 1317 µm, which is around 600 µm less deep than nivolumab. However, the accumulation of ATTO 647N in the epidermis of the AFL-treated skin, might have influenced the biodistribution throughout the skin. The retention in the epidermis might halt ATTO 647N from further diffusion into deeper dermis. The reason for this could be the molecule’s polarity, charge, or possible reactive groups [1]. Topical drug incubation time is also a factor that can have an impact on the amount and depth of the uptake. Several studies show that longer incubation time results in a higher uptake [5,7,15,27,28]. ICG in Nieboer et al. and ATTO 647N have a comparable molecular size, but ICG was only quantifiable in a depth of up to 400 µm. However, in our study, ATTO 647N uptake was analyzed after 20 h of diffusion, while the ICG uptake was measured after 3 h [24]. This emphasizes the importance of incubation time in LADD.

The MFI of AFL-treated skin with topically applied nivolumab or ATTO 647N increased by a factor of 1.61 and 1.43, respectively, compared to the MFI of intact skin. In the literature, however, laser-assisted delivery of large molecules has shown a lower uptake compared with smaller molecules (400 vs. 3350 Da and 350 vs. 5000 Da) [3,20,29]. The direct comparison of the MFI between nivolumab and ATTO 647N is, however, limited as the fluorescence signals originate from two fluorophores with different optical properties and, thus, individual imaging application setups. Furthermore, we did not account for autofluorescence and fluorescence signals from residual compound on the skin surface. Consequently, the resulting ratios of 1.61 and 1.43, are lower than they ought to be. In images of intact skin, a greater autofluorescence, particularly in the hair follicles, is seen in the EVCM images of nivolumab (488 nm), compared with ATTO 647N. Autofluorescence in the 488 nm spectrum is a well-known phenomenon shown in other studies and correction by subtracting the autofluorescent signal via an un-treated control sample could have increased the ratio [6]. In addition, the high fluorescent signal from the remaining ATTO 647N on the skin surface of the intact skin creates a higher MFI and, consequently, a lower ratio. Given the proof-of-concept design, sample size limited statistical evaluation. Thus, if a larger sample size was obtained it would most likely result in a significant higher fluorescent intensity for AFL-assisted uptake of nivolumab compared with intact skin (*n* = 3, +61%), as seen with ATTO 647N (*n* = 4, +43%). In this proof-of-concept study we have demonstrated AFL-assisted delivery ex vivo using minimally invasive settings. Studies exploring optimal device settings for different clinical scenarios, in vivo delivery, and different laser types are warranted. Furthermore, there is a growing interest in the use of micro-/nanoparticles in dermatology and the synergistic use of various drug carrier systems e.g., magnetic-field-assisted delivery, nanoparticles, and physical penetration enhancers, including laser and ultrasound, which would be interesting to explore in this context [30,31,32,33,34]. In addition, future studies on in vivo human skin are necessary for the clinical utility of LADD of therapeutic antibodies using minimally invasive settings.

## 5. Conclusions

In conclusion, shallow laser channels with a median depth of 31.9 µm penetrating less than half-way into the epidermis (median thickness 70.3 µm) were sufficient to enhance transdermal drug delivery. Imaging of the laser-assisted topical uptake of both nivolumab and ATTO 647N revealed a homogenous, horizontal band-like biodistribution pattern that was similar for the two hydrophilic molecules, thus showing that skin biodistribution was independent of molecular size. The uptake for both molecules reached around mid-dermis, with ATTO 647N showing an increased deposition in the epidermis. Further, the results of our study support the conclusion that the compatibility of LADD using minimally invasive settings may not be dependent on molecular size. The use of low pulse energy and density may help decrease procedural discomfort and down-time associated with AFL treatments.

## Figures and Tables

**Table 1:** Fluorescence intensity.

Intervention	*n*	Median MFI (IQR, AU)	*p*-Value
Intact skin + nivolumab	3	1731 (1715/1765)	0.100
AFL + nivolumab	3	2783 (2544/2838)	
Intact skin + ATTO 647N	4	1911 (1811/2048)	0.029
AFL + ATTO 647N	4	2727 (2436/3076)	
Total median	14	-	-

Abbreviations: AFL: ablative fractional laser. MFI: mean fluorescence intensity. IQR: interquartile range. AU: arbitrary unit.

## Data Availability

The data presented in this study are available on request from the corresponding author.

## References

[1] Wenande E., Anderson R.R., Haedersdal M. (2020). Fundamentals of fractional laser-assisted drug delivery: An in-depth guide to experimental methodology and data interpretation. Adv. Drug Deliv. Rev..

[2] Haedersdal M., Erlendsson A.M., Paasch U., Anderson R.R. (2016). Translational medicine in the field of ablative fractional laser (AFXL)-assisted drug delivery: A critical review from basics to current clinical status. J. Am. Acad. Dermatol..

[3] Haak C.S., Bhayana B., Farinelli W.A., Anderson R.R., Haedersdal M. (2012). The impact of treatment density and molecular weight for fractional laser-assisted drug delivery. J. Control. Release Off. J. Control. Release Soc..

[4] Bos J.D., Meinardi M.M.H.M. (2000). The 500 Dalton rule for the skin penetration of chemical compounds and drugs. Exp. Dermatol..

[5] Hendel K.K., Bagger C., Olesen U.H., Janfelt C., Hansen S.H., Haedersdal M., Lerche C.M. (2019). Fractional laser-assisted topical delivery of bleomycin quantified by LC-MS and visualized by MALDI mass spectrometry imaging. Drug Deliv..

[6] Lapteva M., Del Río-Sancho S., Wu E., Carbonell W.S., Böhler C., Kalia Y.N. (2019). Fractional laser ablation for the targeted cutaneous delivery of an anti-CD29 monoclonal antibody-OS2966. Sci. Rep..

[7] Wenande E., Olesen U.H., Nielsen M.M.B., Janfelt C., Hansen S.H., Anderson R.R., Haedersdal M. (2017). Fractional laser-assisted topical delivery leads to enhanced, accelerated and deeper cutaneous 5-fluorouracil uptake. Expert Opin. Drug Deliv..

[8] Olesen U.H., Clergeaud G., Hendel K.K., Yeung K., Lerche C.M., Andresen T.L., Haedersdal M. (2020). Enhanced and Sustained Cutaneous Delivery of Vismodegib by Ablative Fractional Laser and Microemulsion Formulation. J. Investig. Dermatol..

[9] Taudorf E.H., Lerche C.M., Erlendsson A.M., Philipsen P.A., Hansen S.H., Janfelt C., Paasch U., Anderson R.R., Haedersdal M. (2016). Fractional laser-assisted drug delivery: Laser channel depth influences biodistribution and skin deposition of methotrexate. Lasers Surg. Med..

[10] Cao Y., Kakar P., Hossen M.N., Wu M.X., Chen X. (2017). Sustained epidermal powder drug delivery via skin microchannels. J. Control. Release Off. J. Control. Release Soc..

[11] Yu J., Kalaria D.R., Kalia Y.N. (2011). Erbium:YAG fractional laser ablation for the percutaneous delivery of intact functional therapeutic antibodies. J. Control. Release.

[12] Zhang X., Jiang H., Zhang Y., Ren G., Dong L., Zhu J., Tu Y., Tao J., Yang L. (2019). Transdermal delivery of water-soluble fluorescent antibody mediated by fractional Er:YAG laser for the diagnosis of lupus erythematosus in mice. Lasers Surg. Med..

[13] Christensen R.L., Omland S.H., Persson D.P., Husted S., Haedersdal M., Olesen U.H. (2020). Topical Delivery of Nivolumab, a Therapeutic Antibody, by Fractional Laser and Pneumatic Injection. Lasers Surg. Med..

[14] Christensen R.L., Hendel K.K., Persson D.P., Husted S., Olesen U.H., Haedersdal M. (2021). Topical delivery of PD-1 inhibitors with laser-assisted passive diffusion and active intradermal injection: Investigation of cutaneous pharmacokinetics and biodistribution patterns. Lasers Surg. Med..

[15] Wenande E., Olesen U.H., Boesen M.R., Persson D.P., Lerche C.M., Stürup S., Gammelgaard B., Husted S., Anderson R.R., Haedersdal M. (2018). Laser-assisted delivery enhances topical uptake of the anticancer agent cisplatin. Drug Deliv..

[16] Bağcı I.S., Aoki R., Vladimirova G., Sárdy M., Ruzicka T., French L.E., Hartmann D. (2021). Simultaneous immunofluorescence and histology in pemphigus vulgaris using ex vivo confocal laser scanning microscopy. J. Biophotonics.

[17] Sattler E.C., Kästle R., Welzel J. (2013). Optical coherence tomography in dermatology. J. Biomed. Opt..

[18] Schindelin J., Arganda-Carreras I., Frise E., Kaynig V., Longair M., Pietzsch T., Preibisch S., Rueden C., Saalfeld S., Schmid B. (2012). Fiji-an Open Source platform for biological image analysis. Nat. Methods.

[19] Banzhaf C.A., Ortner V.K., Philipsen P.A., Haedersdal M. (2019). The ablative fractional coagulation zone influences skin fluorescence intensities of topically applied test molecules-An in vitro study with fluorescence microscopy and fluorescence confocal microscopy. Lasers Surg. Med..

[20] Haak C.S., Hannibal J., Paasch U., Anderson R.R., Haedersdal M. (2017). Laser-induced thermal coagulation enhances skin uptake of topically applied compounds. Lasers Surg. Med..

[21] Paasch U., Haedersdal M. (2011). Laser systems for ablative fractional resurfacing. Expert Rev. Med. Devices.

[22] Meesters A.A., Nieboer M.J., Almasian M., Georgiou G., de Rie M.A., Verdaasdonk R.M., Wolkerstorfer A. (2019). Drug penetration enhancement techniques in ablative fractional laser assisted cutaneous delivery of indocyanine green. Lasers Surg. Med..

[23] Chen X., Shah D., Kositratna G., Manstein D., Anderson R.R., Wu M.X. (2012). Facilitation of transcutaneous drug delivery and vaccine immunization by a safe laser technology. J. Control. Release.

[24] Nieboer M.J., Meesters A.A., Almasian M., Georgiou G., de Rie M.A., Verdaasdonk R.M., Wolkerstorfer A. (2020). Enhanced topical cutaneous delivery of indocyanine green after various pretreatment regimens: Comparison of fractional CO2 laser, fractional Er:YAG laser, microneedling, and radiofrequency. Lasers Med. Sci..

[25] Rosenberg L.K., Bagger C., Janfelt C., Haedersdal M., Olesen U.H., Lerche C.M. (2021). A Comparison of Human and Porcine Skin in Laser-Assisted Drug Delivery of Chemotherapeutics. Lasers Surg. Med..

[26] Kositratna G., Hibert M.L., Jaspan M., Welford D., Manstein D. (2016). Effects of deviation from focal plane on lesion geometry for ablative fractional photothermolysis. Lasers Surg. Med..

[27] Olesen U.H., Clergeaud G., Lerche C.M., Andresen T.L., Haedersdal M. (2019). Topical delivery of vismodegib using ablative fractional laser and micro-emulsion formulation in vitro: Topical vismodegib and laser in vitro. Lasers Surg. Med..

[28] Haak C.S., Christiansen K., Erlendsson A.M., Taudorf E.H., Thaysen-Petersen D., Wulf H.C., Haedersdal M. (2016). Ablative fractional laser enhances MAL-induced PpIX accumulation: Impact of laser channel density, incubation time and drug concentration. J. Photochem. Photobiol. B.

[29] Zorec B., Škrabelj D., Marinček M., Miklavčič D., Pavšelj N. (2017). The effect of pulse duration, power and energy of fractional Er:YAG laser for transdermal delivery of differently sized FITC dextrans. Int. J. Pharm..

[30] Ryzhkov A., Raikher Y. (2019). Size-Dependent Properties of Magnetosensitive Polymersomes: Computer Modelling. Sensors.

[31] Kurlyandskaya G.V., Blyakhman F.A., Makarova E.B., Buznikov N.A., Safronov A.P., Fadeyev F.A., Shcherbinin S.V., Chlenova A.A. (2020). Functional magnetic ferrogels: From biosensors to regenerative medicine. AIP Adv..

[32] Kennedy S., Roco C., Déléris A., Spoerri P., Cezar C., Weaver J., Vandenburgh H., Mooney D. (2018). Improved magnetic regulation of delivery profiles from ferrogels. Biomaterials.

[33] Gambin B., Kruglenko E., Tymkiewicz R., Litniewski J. (2019). Ultrasound assessment of the conversion of sound energy into heat in tissue phantoms enriched with magnetic micro- and nanoparticles. Med. Phys..

[34] Patra J.K., Das G., Fraceto L.F., Campos E.V.R., Rodriguez-Torres M.d.P., Acosta-Torres L.S., Diaz-Torres L.A., Grillo R., Swamy M.K., Sharma S. (2018). Nano based drug delivery systems: Recent developments and future prospects. J. Nanobiotechnology.

